# Supplementary material for: SAMHD1 is a key regulator of the lineage-specific response of acute lymphoblastic leukaemias to nelarabine
Source: Commun Biol. 2020 Jun 24;3:324. doi: 10.1038/s42003-020-1052-8 (PMC7314829; doi:10.1038/s42003-020-1052-8)
Supplement: Supplementary file 1 — Supplementary Information [file 42003_2020_1052_MOESM1_ESM.pdf]

Suppl. Figure 1

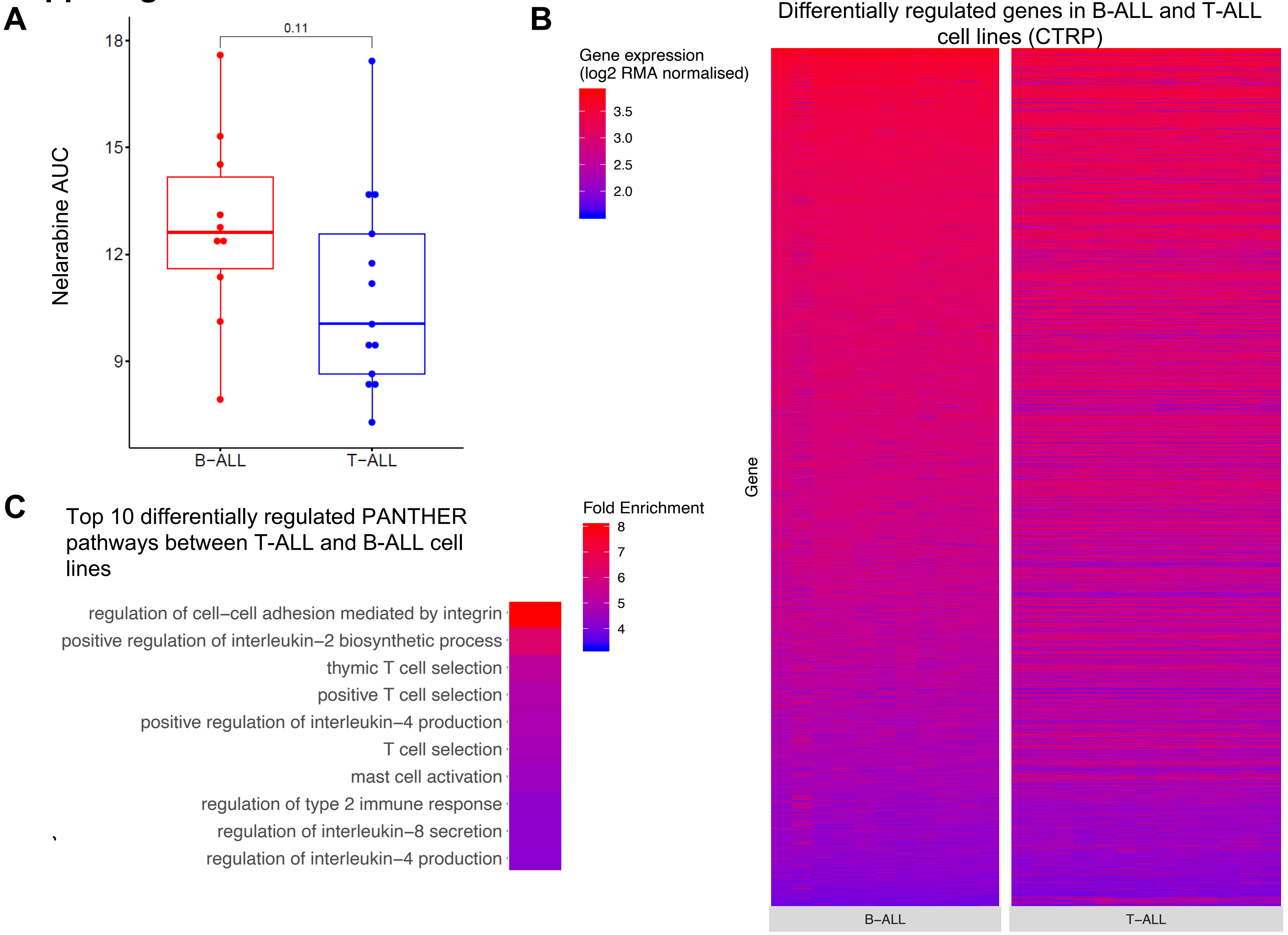

Suppl. Figure 1. Gene expression profiles and nelarabine sensitivity in acute lymphoblastic leukaemia (ALL) cell lines. A) Nelarabine sensitivity expressed as area under the curve (AUC) in T-cell precursor ALL (T-ALL) and B-ALL cell lines from CTRP. B) Heatmap illustrating expression patterns of genes differentially regulated between T-ALL and B-ALL cell lines based on CTRP data. Heatmaps displaying the expression of all genes in the CTRP ALL cell lines and those displaying gene expression in the ALL cell lines in the CCLE and GDSC datasets are provided in Suppl. Figure 1D-H. Individual gene expression values are presented in Suppl. Data 1.

Suppl. Figure 1
D

Heatmap illustrating expression (mRNA abundance) of all genes in B- vs. T-ALL cells based on CTRP data.

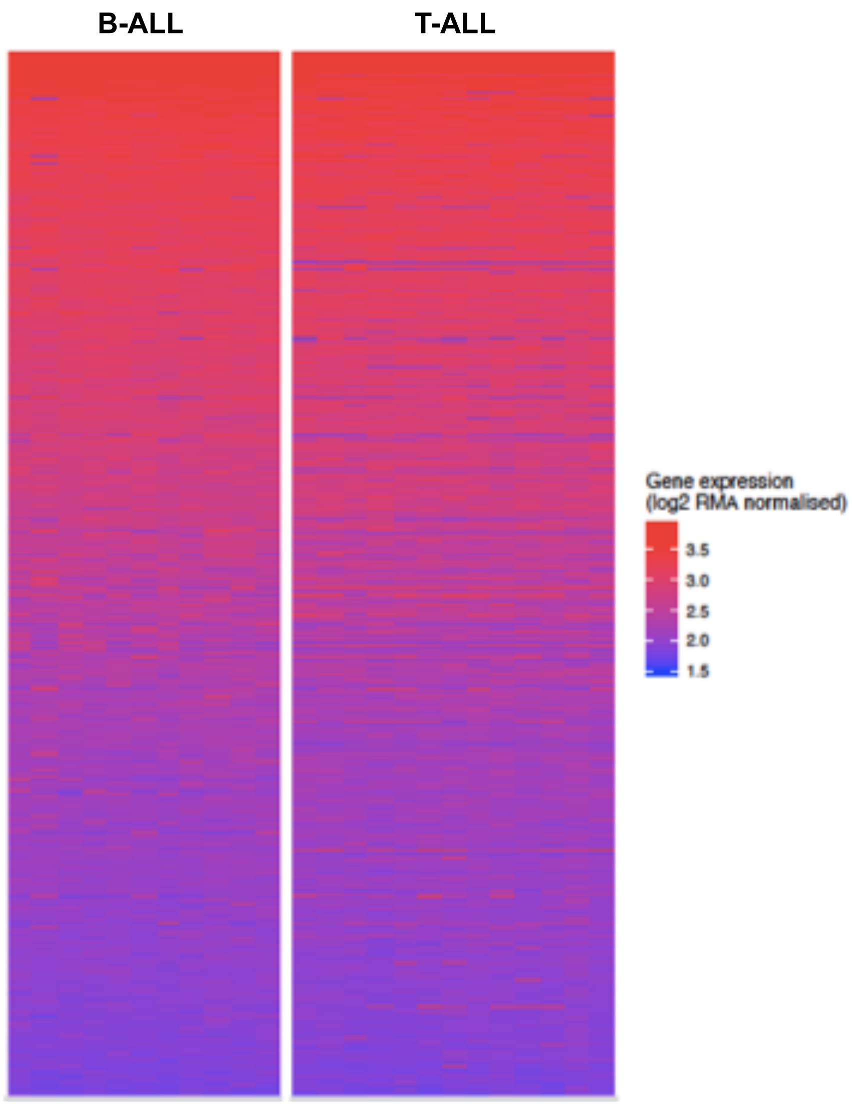

Suppl. Figure 1

E

Heatmap illustrating expression (mRNA abundance) of differentially regulated genes in B- vs. T-ALL cells based on CCLE data.

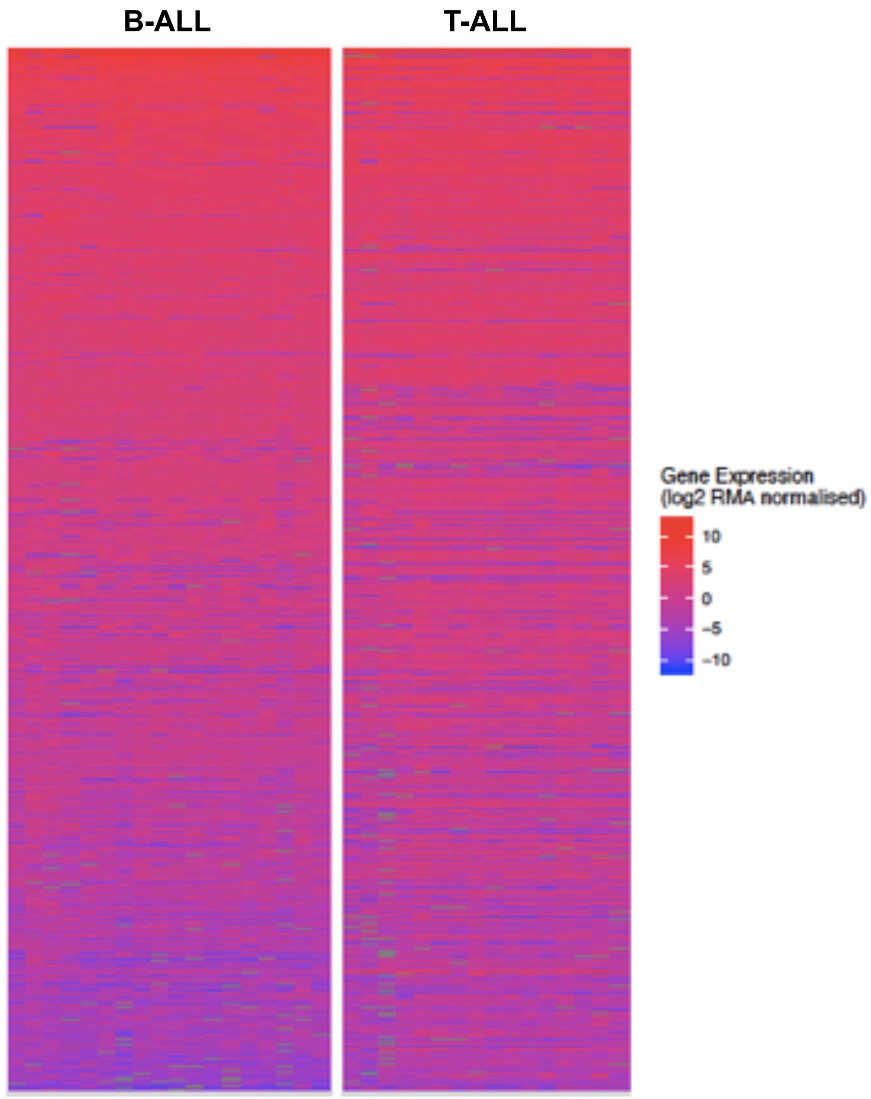

Suppl. Figure 1

F

Heatmap illustrating expression (mRNA abundance) of all genes in B- vs. T-ALL cells based on CCLE data.

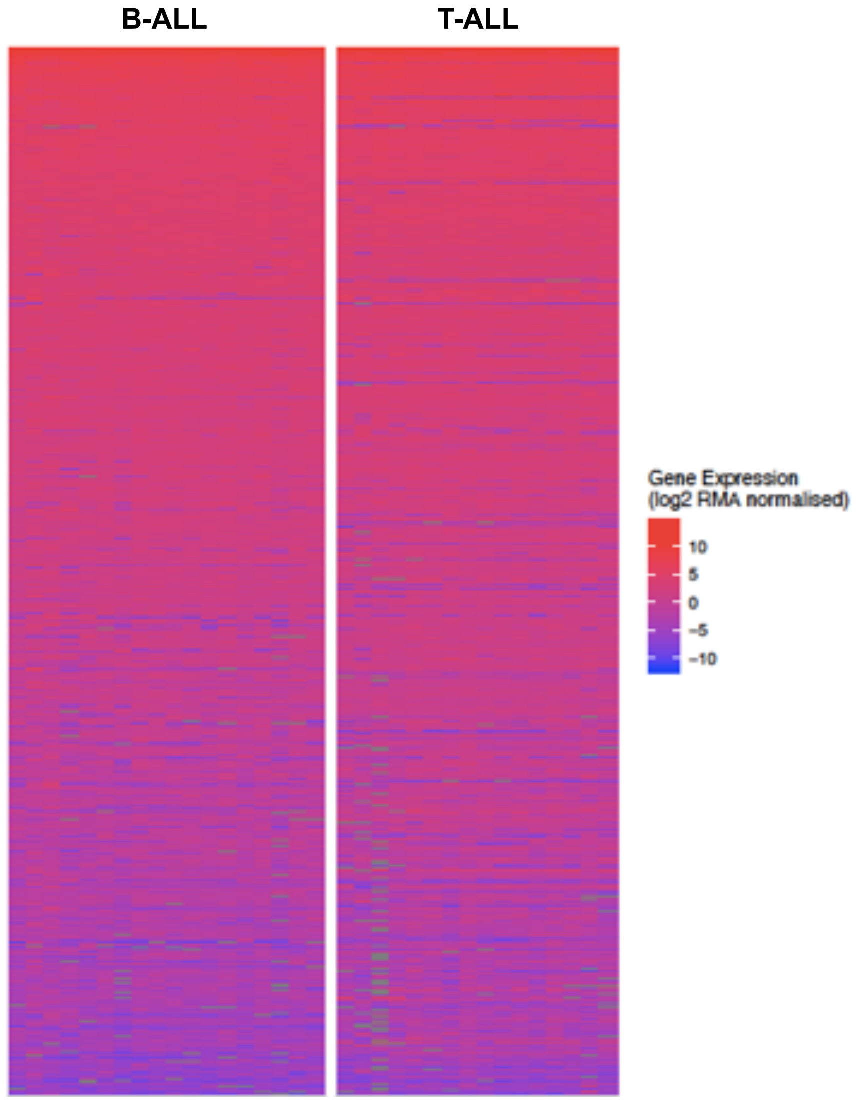

Suppl. Figure 1

G

Heatmap illustrating expression (mRNA abundance) of differentially regulated genes in B- vs. T-ALL cells based on GDSC data.

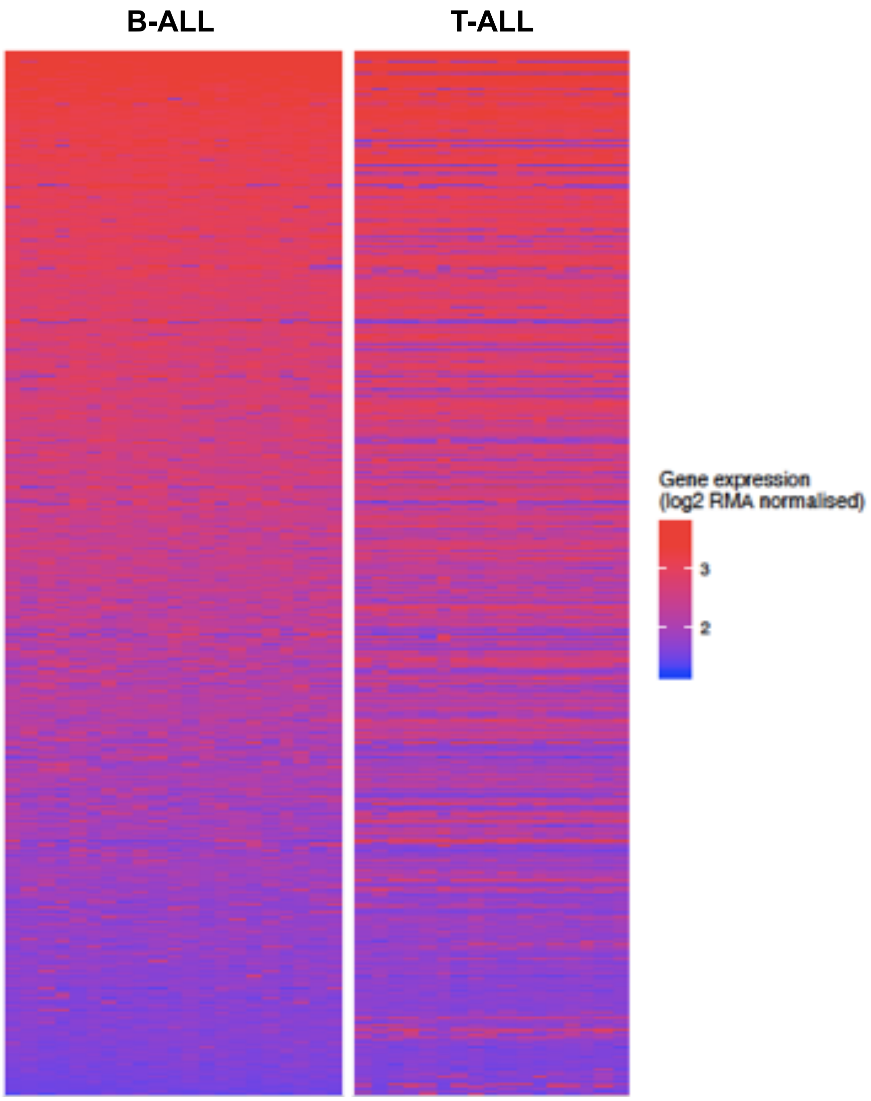

Suppl. Figure 1

H

Heatmap illustrating expression (mRNA abundance) of all genes in B- vs. T-ALL cells based on GDSC data.

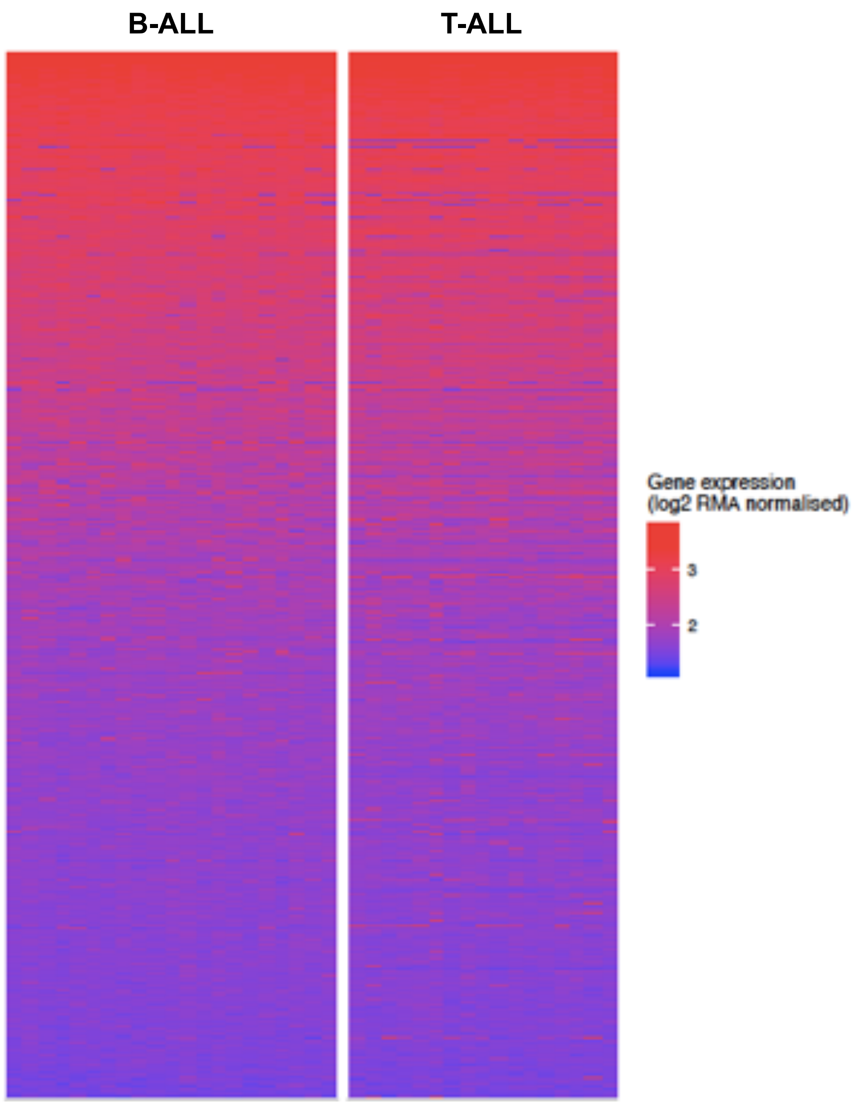

Suppl. Figure 2

A

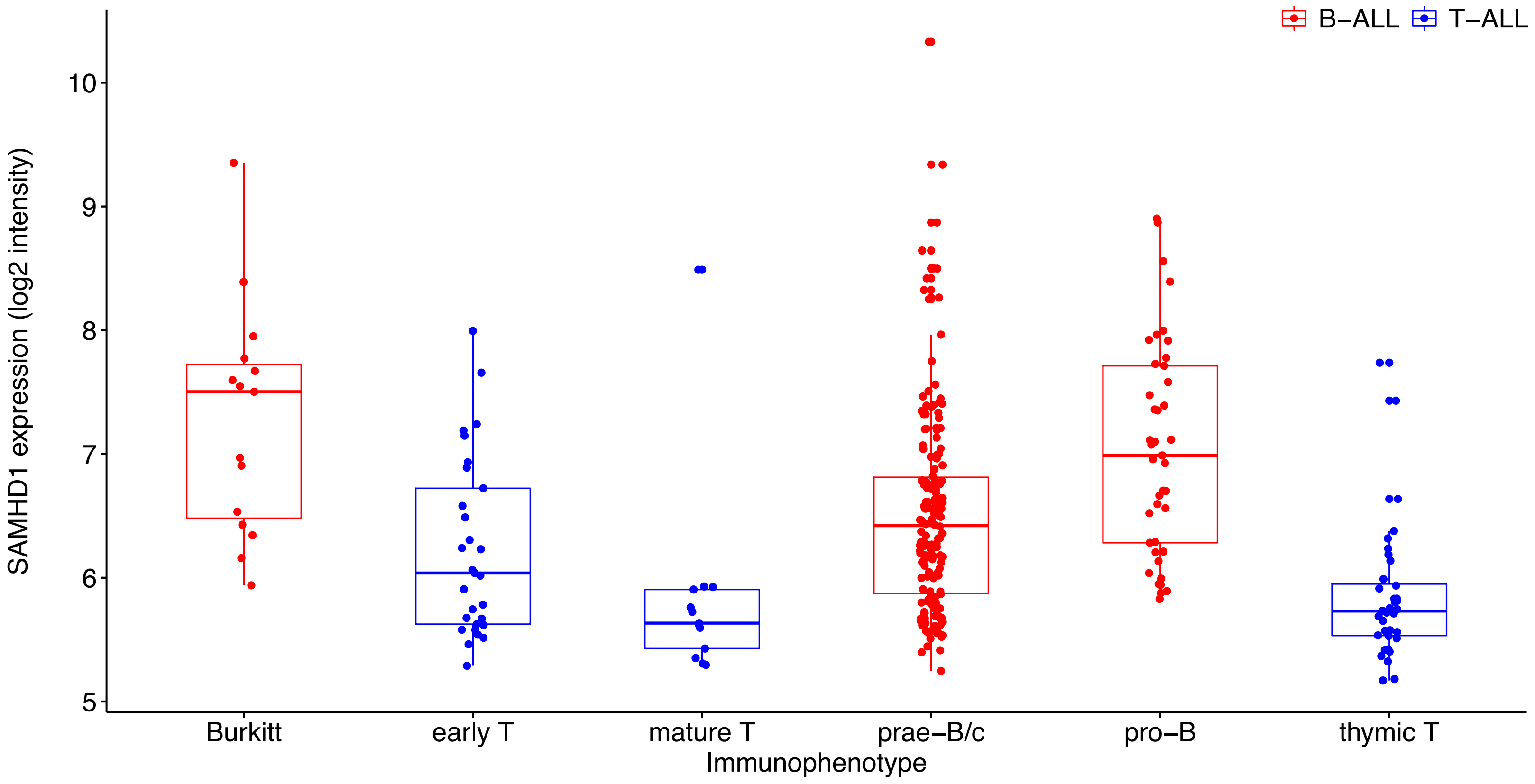

Suppl. Figure 2A. SAMHD1 expression in ALL patients with different immunophenotypes.

	P value
pre-B/ c-Burkitt	0.0067358
early T/ Burkitt	0.00073591
pro-B/ Burkitt	0.927323943
mature T/ Burkitt	5.00E-05
thy T-Burkitt	2.23E-07
early T/ pre-B/c	0.478671852
pro-B/ pre-B/c	0.00285102
mature T/ pre-B/c	0.040886675
thy T/ pre-B/c	9.73E-05
pro-B/ early T	0.000553081
mature T/ early T	0.68842299
thy T/ early T	0.379143369
mature T/ pro-B	5.40E-05
thy T/ pro-B	3.67E-09
thy T/ mature T	1

Suppl. Figure 2A. SAMHD1 expression in ALL patients with different immunophenotypes. *P* values for comparisons between individual groups.

Suppl. Figure 2
B

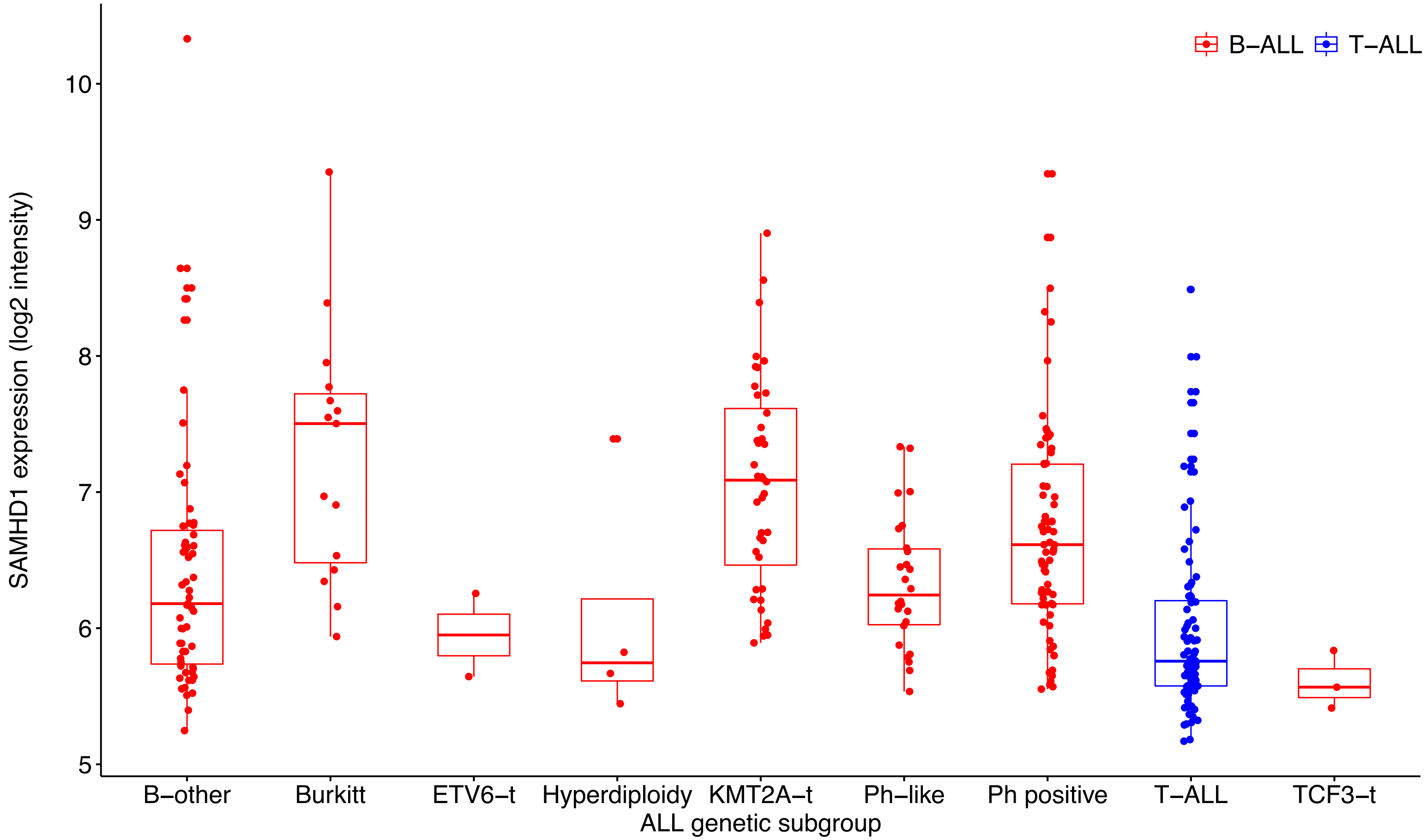

Suppl. Figure 2B. SAMHD1 expression in ALL patients with different genotypes.

	P value
2 vs. 1	0.38282375
3 vs. 1	0.999905
4 vs. 1	0.00230736
5 vs. 1	0.02880067
6 vs. 1	0.00619649
7 vs. 1	0.70123101
8- vs. 1	0.99542401
9 vs. 1	0.99530362
3 vs. 2	0.38981245
4 vs. 2	0.41595306
5 vs. 2	2.73E-07
6 vs. 2	0.26166224
7 vs. 2	0.2631177
8 vs. 2	0.8994844
9 vs. 2	0.79514518
4 vs. 3	0.00682233
5 vs. 3	0.55549758
6 vs. 3	0.0073478
7 vs. 3	0.84170864
8 vs. 3	0.99912995
9 vs. 3	0.99961577

	P value
5 vs. 4	1.60E-10
6 vs. 4	0.99429435
7 vs. 4	0.04940224
8 vs. 4	0.56219644
9 vs. 4	0.28423458
6 vs. 5	3.79E-07
7 vs. 5	0.99596858
8 vs. 5	1
9 vs. 5	0.99999962
7 vs. 6	0.02309725
8 vs. 6	0.37573601
9 vs. 6	0.14840814
8 vs. 7	0.99992001
9 vs. 7	0.99681029
9 vs. 8	0.99999994

Suppl. Figure 2B. SAMHD1 expression in ALL patients with different genotypes. *P* values for comparisons between individual groups (1=B-other, 2=Ph pos, 3=Ph-like, 4=KMT2A, 5=T-ALL, 6=Burkitt, 7=TCF3, 8=ETV6, 9=Hyperdip.

Suppl. Figure 3

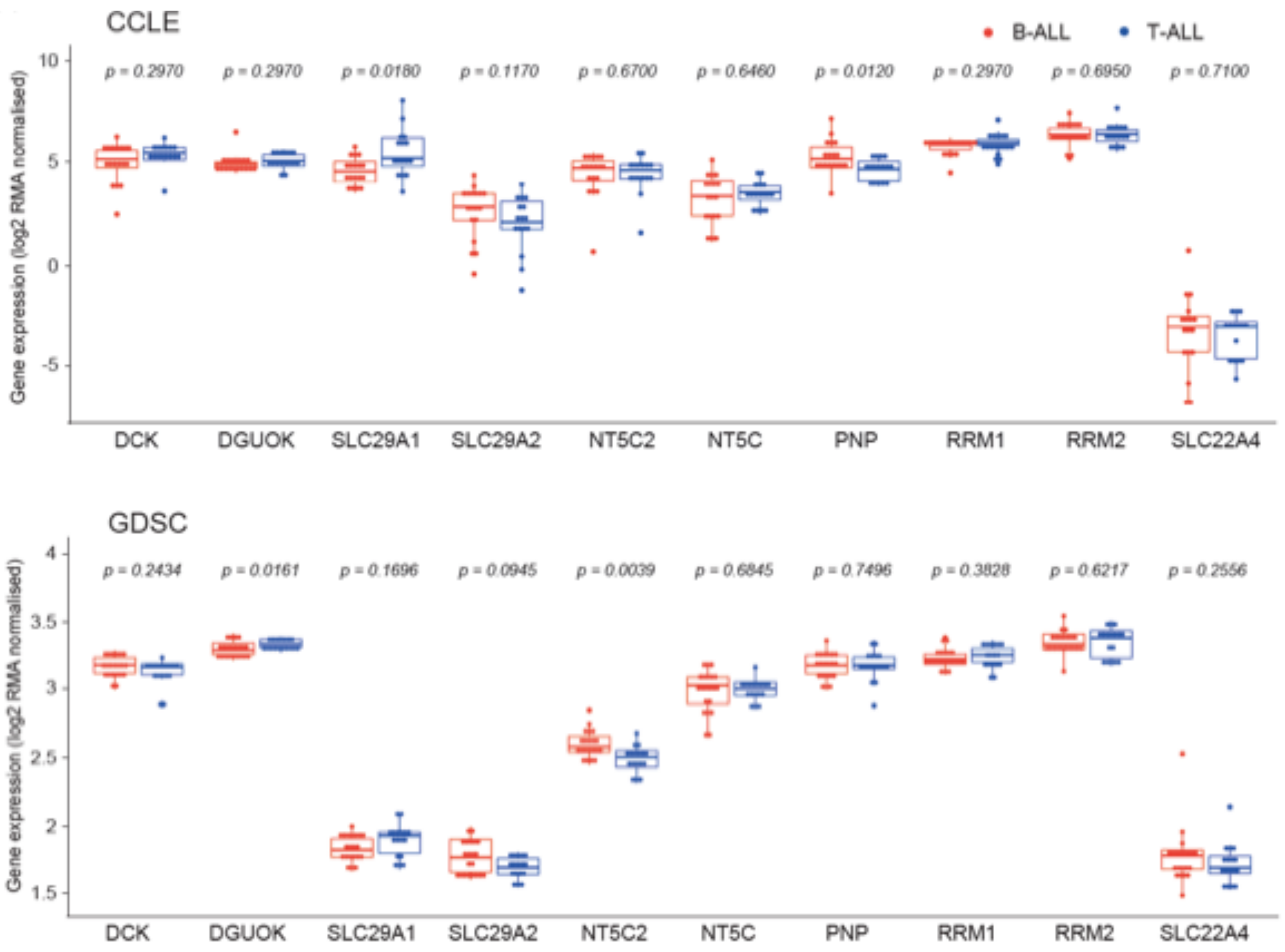

Suppl. Figure 3. Expression of genes known to be potentially involved in nucleoside analogue activity in B-ALL and T-ALL cell lines in the CCLE and GDSC.

Suppl. Figure 3

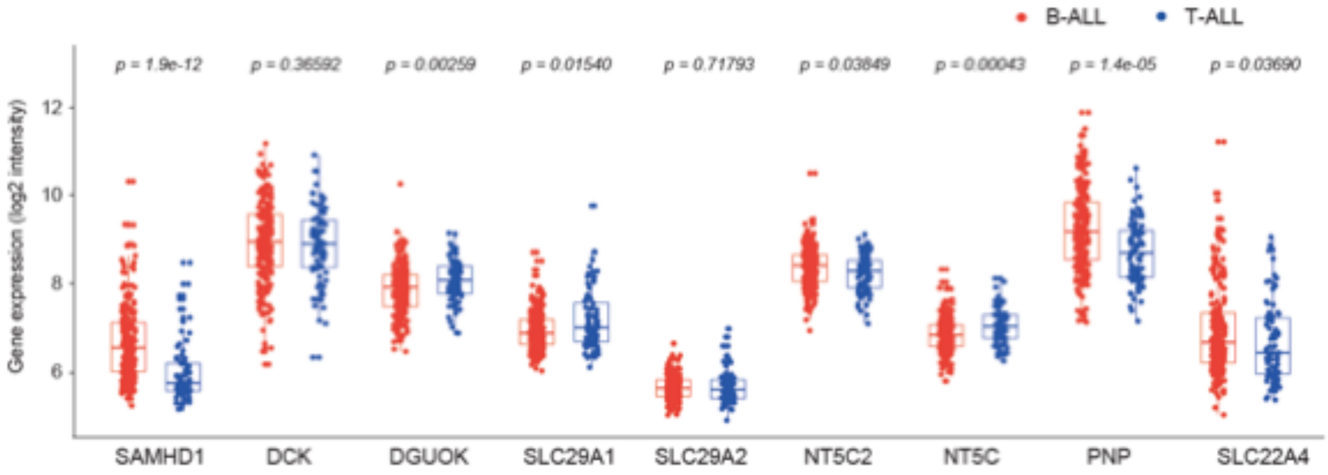

Suppl. Figure 3. Expression of genes known to be potentially involved in nucleoside analogue activity in patient-derived B-ALL and T-ALL cells.

Suppl. Figure 4

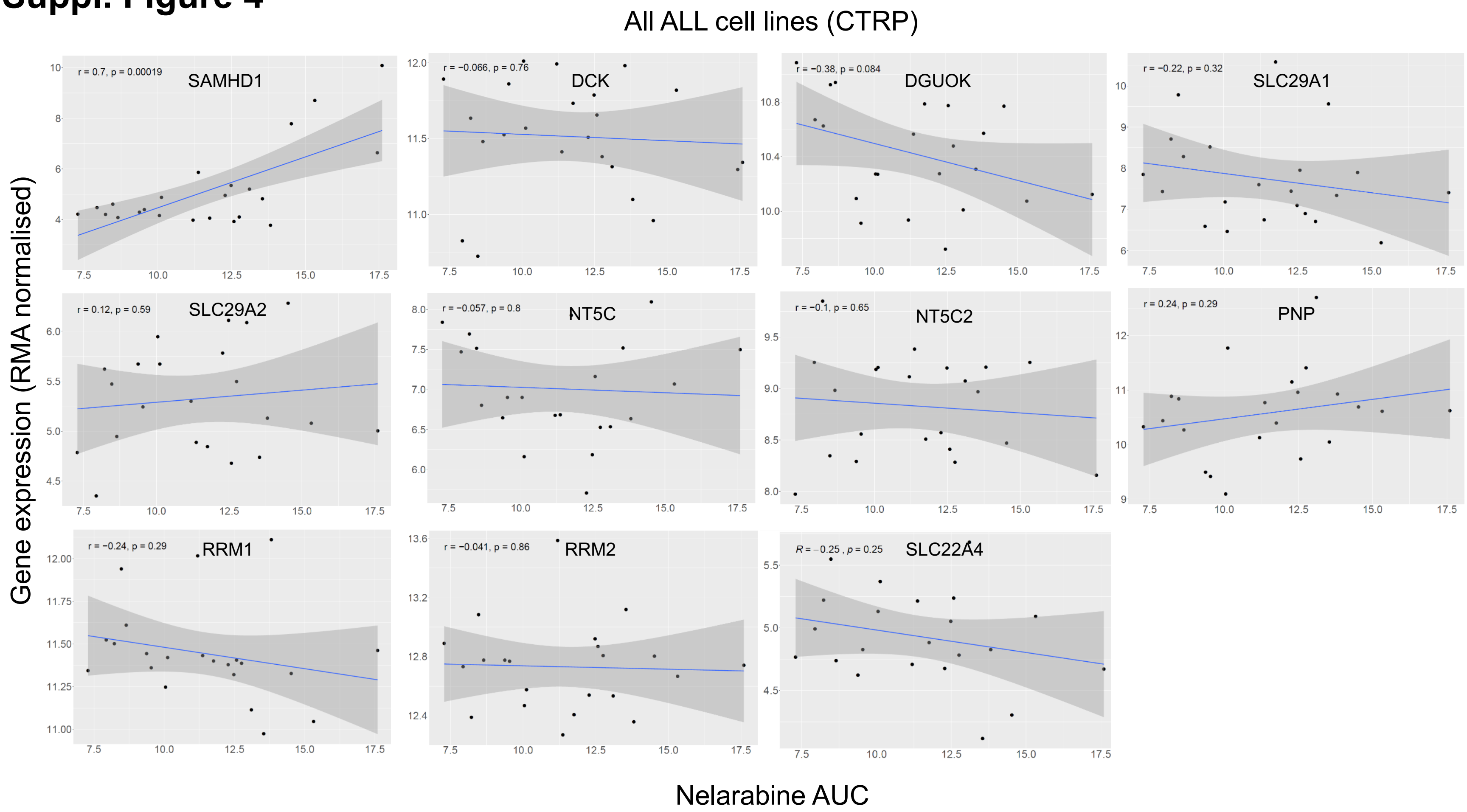

Suppl. Figure 4

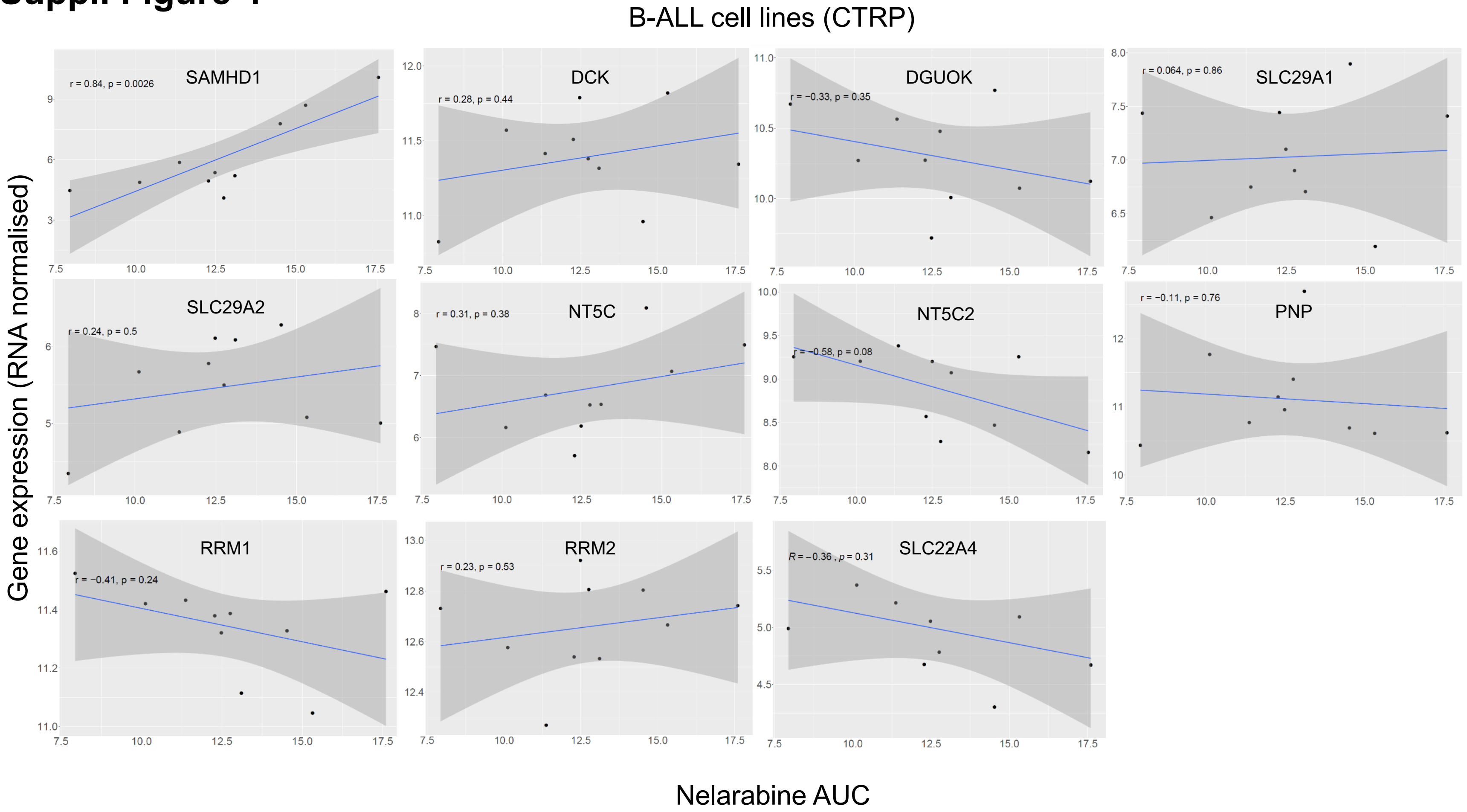

Suppl. Figure 4

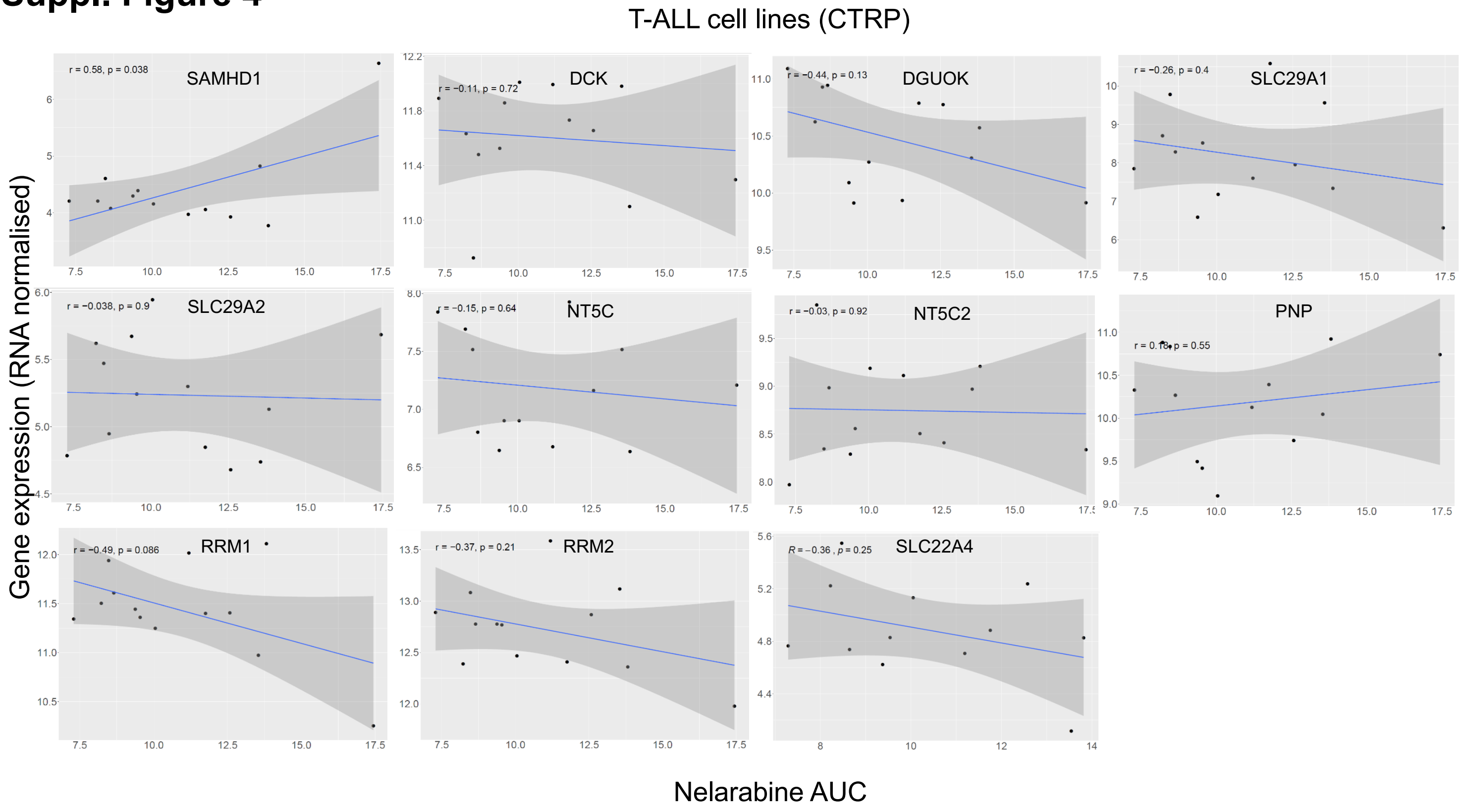

Suppl. Figure 4. Correlation of the expression of genes (mRNA abundance) known to affect nucleoside analogue activity to the nelarabine sensitivity (expressed as AUC) across all ALL, the B-ALL and the T-ALL cell lines based on CTRP data. Pearson’s r values and respective p-values are provided.

Suppl. Figure 5

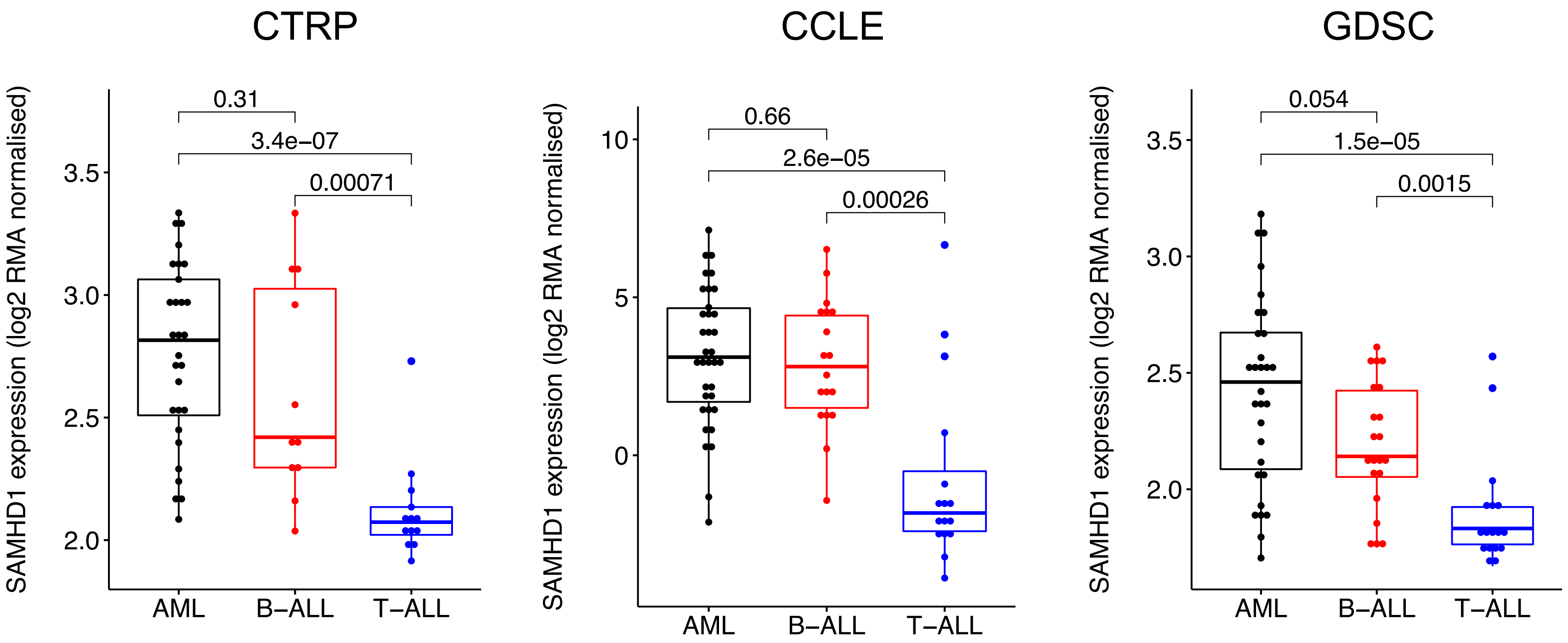

Suppl. Figure 5. Comparison of SAMHD1 expression (mRNA abundance) levels in acute myeloid leukaemia (AML), B-cell acute lymphoblastic leukaemia (B-ALL), T-cell acute lymphoblastic leukaemia (T-ALL) cells in CTRP, CCLE, and GDSC. Respective p-values are provided.

Suppl. Figure 6

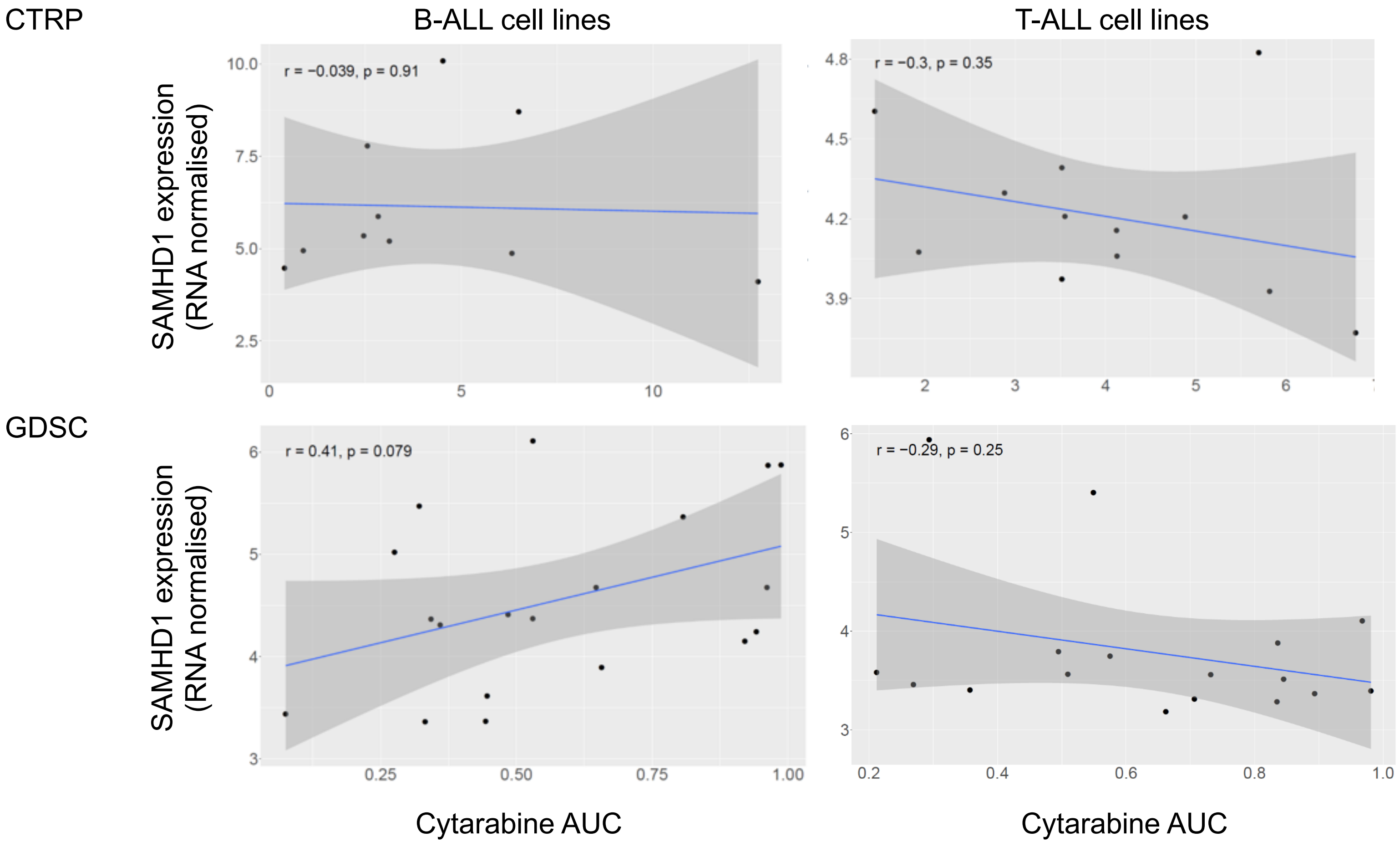

Suppl. Figure 6. Correlations of SAMHD1 expression (mRNA abundance) with the cytarabine AUC exclusively in B-ALL- and T-ALL cell lines based on CTRP and GDSC data. Pearson's r values and respective p-values are provided.

Suppl. Figure 7

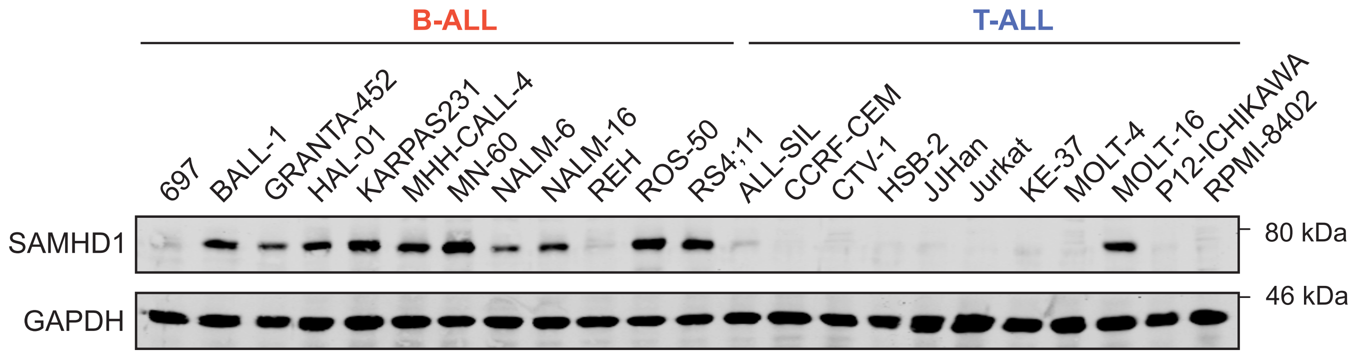

For comparison:
Figure 3A

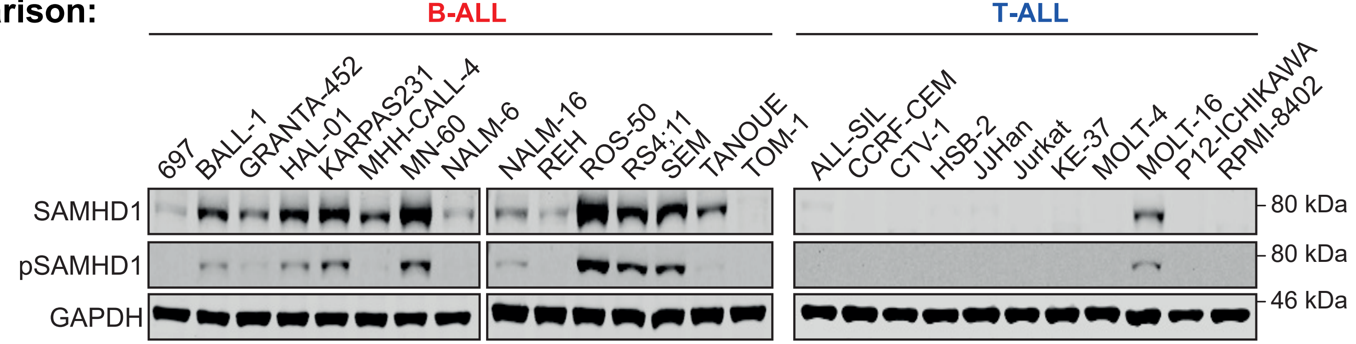

Suppl. Figure 7. SAMHD1 protein levels in the RCCL panel of B-ALL and T-ALL cell lines. Representative Western blots indicating protein levels of total SAMHD1 and GAPDH in 23 cell lines of the RCCL panel, which were run on the same gel and blotted on the same membrane to confirm the representativeness of the blots provided in Figure 3A. Figure 3A is provided for comparison.

Suppl. Figure 8

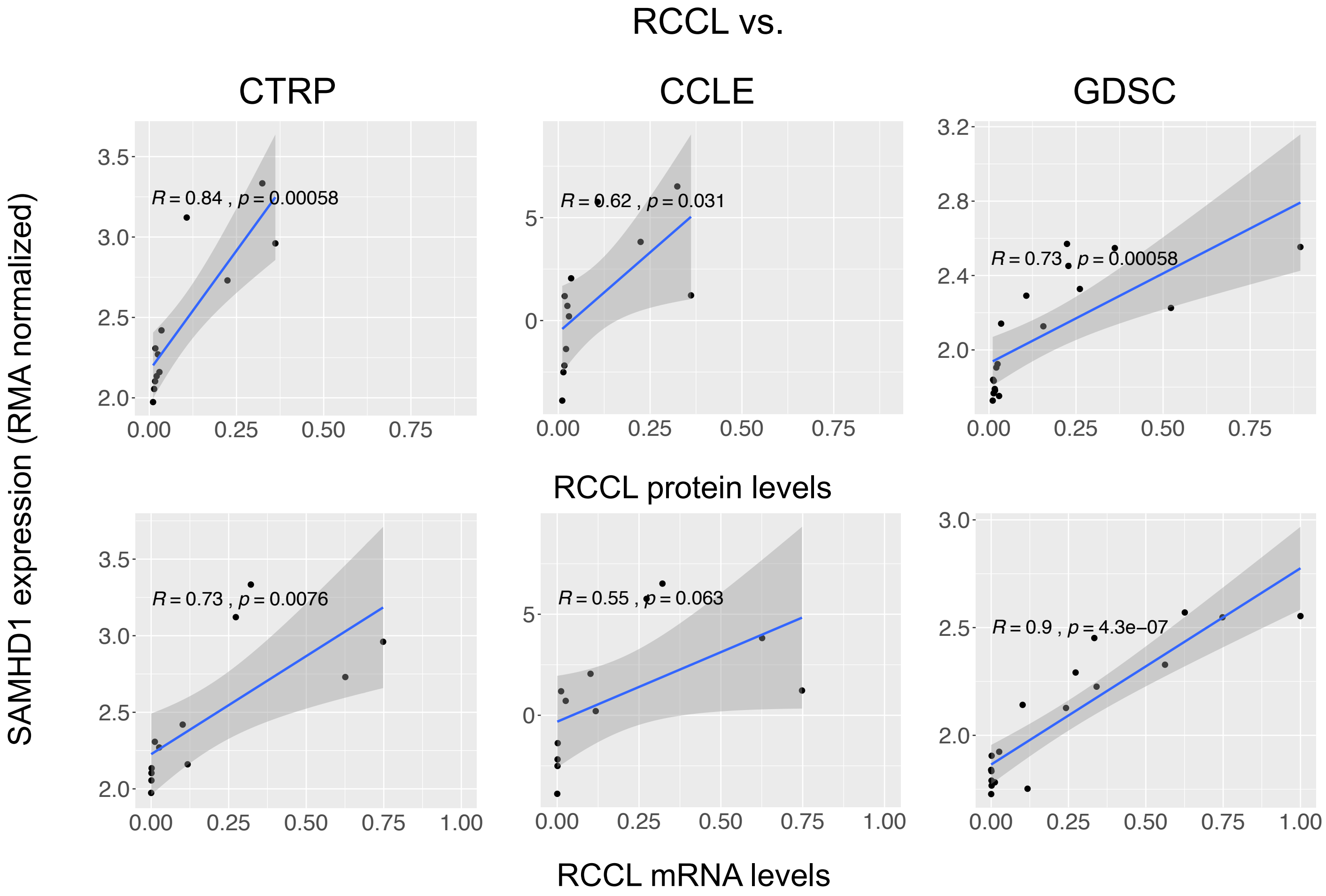

Suppl. Figure 8. Correlations of SAMHD1 protein and mRNA levels determined in the RCCL cell lines with the SAMHD1 expression data derived from the CTRP, CCLE, and GDSC among the cell lines that are represented in both respective datasets. Pearson's r values and respective p-values are provided.

Suppl. Figure 9

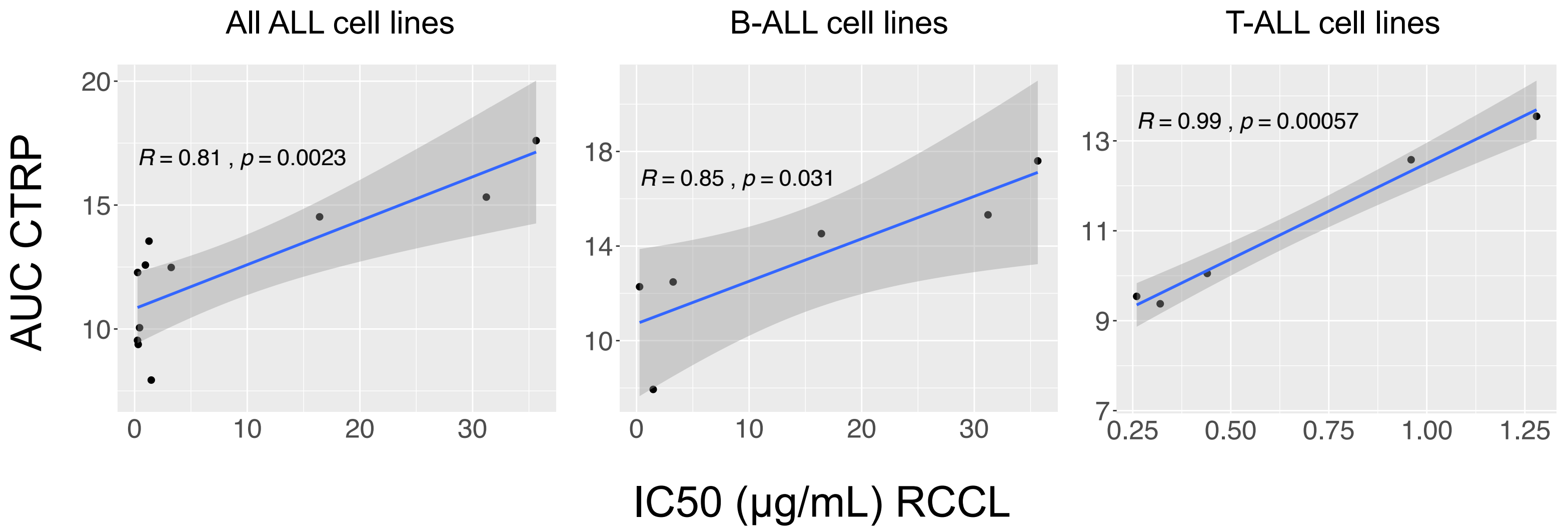

Suppl. Figure 9. Correlations of the nelarabine AUCs derived from the CTRP and the AraG IC50 values determined in the RCCL panel across the ALL cell lines present in both datasets. Pearson's r values and respective p-values are provided.

Suppl. Figure 10

Figure 3

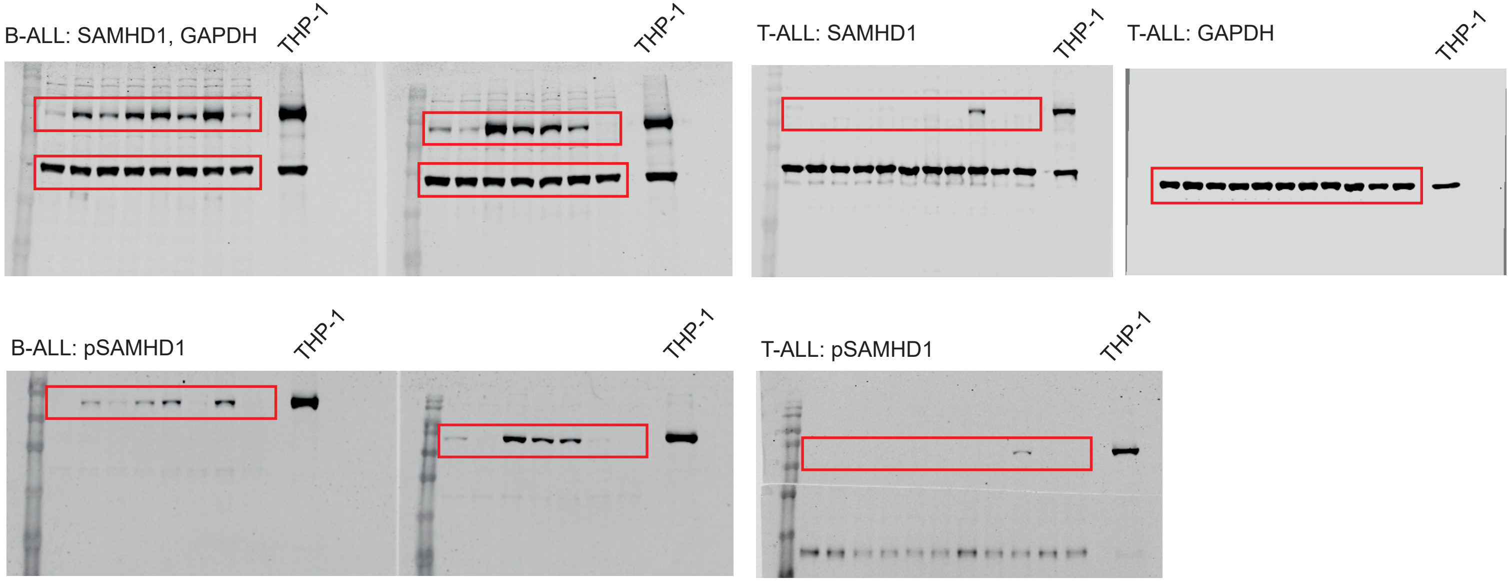

Figure 5A

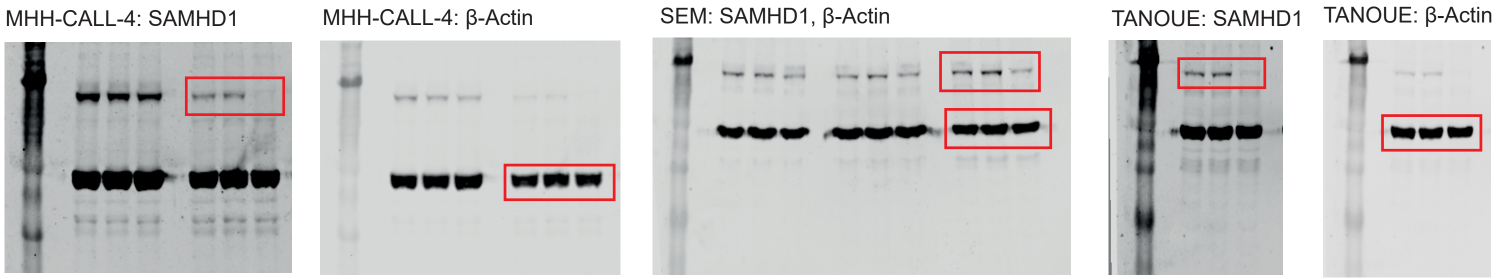

Suppl. Figure 10. Uncropped Western blots and agarose gels.

Figure 5B

THP-1: SAMHD1, β-Actin

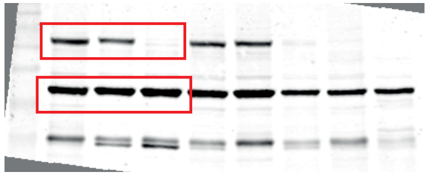

HEL: SAMHD1, β-Actin

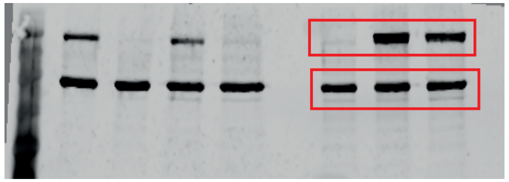

Jurkat: SAMHD1

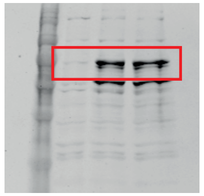

Jurkat: β-Actin

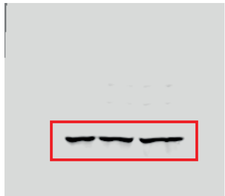

Suppl. Figure 8

SAMHD1

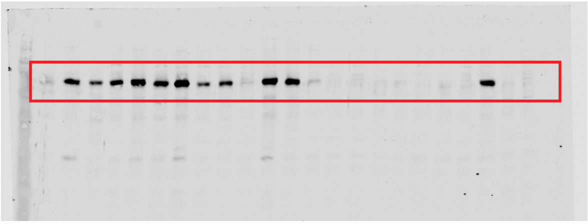

GAPDH

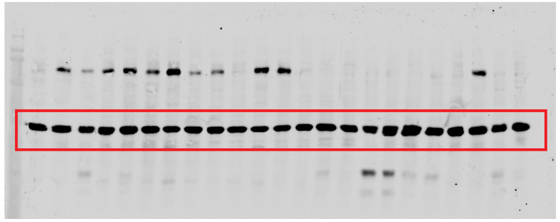

Suppl. Figure 10. Uncropped Western blots and agarose gels.

Figure 6A

SAMHD1

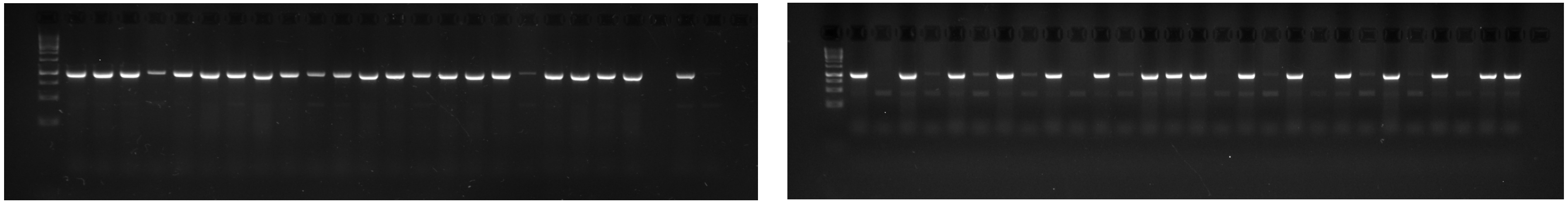

GAPDH

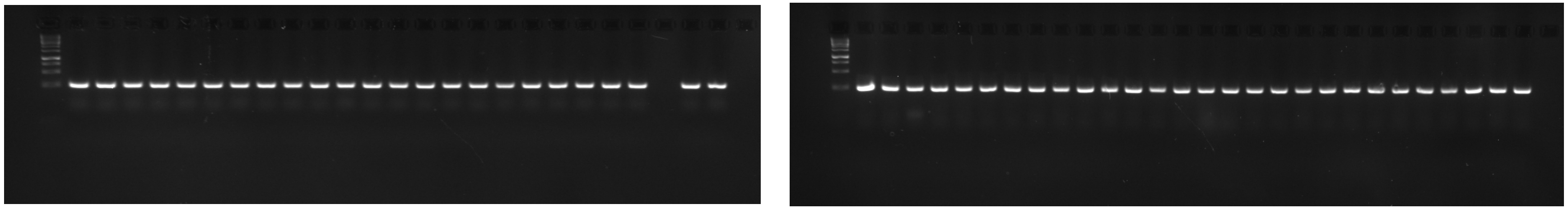

Suppl. Figure 10. Uncropped Western blots and agarose gels.

Suppl. Table 1. B-ALL and T-ALL cell lines in the CCLE and GDSC (overlaps highlighted in *italics*).

CCLE		GDSC	
**Cell Line**	**Lineage**	**Cell line**	**Lineage**
697	B-ALL	*697*	B-ALL
A4FUK	B-ALL	ALL-PO	B-ALL
EHEB	B-ALL	BALL-1	B-ALL
HUNS1	B-ALL	GR-ST	B-ALL
JM-1	B-ALL	HAL-01	B-ALL
KASUMI-2	B-ALL	KARPAS-231	B-ALL
KOPN-8	B-ALL	*KOPN-8*	B-ALL
MHH-CALL2	B-ALL	LC4-1	B-ALL
MHH-CALL3	B-ALL	*MHH-CALL-2*	B-ALL
MHH-CALL4	B-ALL	*MHH-CALL-4*	B-ALL
MUTZ-5	B-ALL	MHH-PREB-1	B-ALL
NALM-19	B-ALL	MN-60	B-ALL
NALM-6	B-ALL	*NALM-6*	B-ALL
RCH-ACV	B-ALL	P30-OHK	B-ALL
REH	B-ALL	*RCH-ACV*	B-ALL
RS-411	B-ALL	*REH*	B-ALL
SEM	B-ALL	ROS-50	B-ALL
SUP-B15	B-ALL	*RS4-11*	B-ALL
		SUP-B15	B-ALL
		SUP-B8	B-ALL
		U-698-M	B-ALL
ALL-SIL	T-ALL	*ALL-SIL*	T-ALL
C8166	T-ALL	ATN-1	T-ALL
DND-41	T-ALL	BE-13	T-ALL
HPB-ALL	T-ALL	CCRF-CEM	T-ALL
JURKAT	T-ALL	*DND-41*	T-ALL
KE-37	T-ALL	HH	T-ALL
LOUCY	T-ALL	*JURKAT*	T-ALL
MOLT-13	T-ALL	KARPAS-45	T-ALL
MOLT-16	T-ALL	*KE-37*	T-ALL
MOLT-3	T-ALL	*LOUCY*	T-ALL
P12-ICHIKAWA	T-ALL	*MOLT-13*	T-ALL
PEER	T-ALL	*MOLT-16*	T-ALL
PF-382	T-ALL	MOLT-4	T-ALL
RPMI-8402	T-ALL	*P12-ICHIKAWA*	T-ALL
SUP-T11	T-ALL	*PF-382*	T-ALL
TALL-1	T-ALL	*RPMI-8402*	T-ALL
		SUP-T11	T-ALL

Suppl. Table 2. B-ALL and T-ALL cell line sensitivity to nelarabine expressed as area under the curve (AUC) derived from CTRP.

**Cell line**	**Lineage**	**AUC**
HPBALL	T-ALL	11.757
DND-41	T-ALL	7.29
SUPT-1	T-ALL	8.4742
JURKAT	T-ALL	12.58
PEER	T-ALL	13.818
PF-382	T-ALL	11.193
ALL-SIL	T-ALL	13.546
P12-ICHIKAWA	T-ALL	9.5418
RPMI-8402	T-ALL	10.052
MOLT-16	T-ALL	17.44
MOLT-13	T-ALL	8.6473
TALL-1	T-ALL	8.2253
KE-37	T-ALL	9.3765
SEM	B-ALL	17.602
RCH-ACV	B-ALL	12.759
MHH-CALL3	B-ALL	10.123
RS-411	B-ALL	14.526
MHH-CALL4	B-ALL	15.322
REH	B-ALL	7.9391
697	B-ALL	12.28
SUP-B15	B-ALL	11.372
KASUMI-2	B-ALL	13.107
NALM-6	B-ALL	12.483

Suppl. Table 3. AraG and cytarabine concentrations that reduce B-ALL and T-ALL cell line sensitivity by 50% (IC50).

**B-ALL**	**IC50**	
**Cell line**	**AraG (µg/mL)**	**Cytarabine (ng/mL)**
697	0.27 ± 0.01	1.23 ± 0.05
BALL-1	4.76 ± 0.50	2.74 ± 0.05
GRANTA-452	6.69 ± 0.70	3.42 ± 0.25
HAL-01	2.15 ± 0.49	0.98 ± 0.04
KARPAS231	26.73 ± 2.62	11.57 ± 0.77
MHH-CALL-4	31.22 ± 2.50	27.07 ± 3.05
MN-60	99.10 ± 1.62	14.95 ± 0.99
NALM-6	3.25 ± 0.28	2.19 ± 0.06
NALM-16	65.19 ± 2.72	8.79 ± 0.42
REH	1.48 ± 0.07	1.31 ± 0.17
ROS-50	90.62 ± 9.05	18.28 ± 3.57
RS4;11	16.42 ± 1.32	5.74 ± 1.89
SEM	35.64 ± 3.71	27.35 ± 3.18
TANOUE	49.14 ± 2.95	27.67 ± 0.60
TOM-1	0.10 ± 0.01	1.38 ± 0.03
**T-ALL**	**IC50**	
**Cell line**	**AraG (µg/mL)**	**Cytarabine (ng/mL)**
ALL-SIL	1.28 ± 0.16	5.17 ± 0.64
CCRF-CEM	0.43 ± 0.02	3.88 ± 0.70
CTV-1	0.38 ± 0.07	1.72 ± 0.01
HSB-2	0.52 ± 0.05	3.51 ± 0.07
JJHan	0.97 ± 0.19	5.61 ± 0.53
Jurkat	0.96 ± 0.06	6.65 ± 0.52
KE-37	0.32 ± 0.11	1.83 ± 0.25
MOLT-4	0.46 ± 0.01	3.02 ± 0.10
MOLT-16	15.55 ± 1.30	6.48 ± 0.53
P12-ICHIKAWA	0.26 ± 0.01	2.12 ± 0.19
RPMI-8402	0.44 ± 0.01	3.43 ± 0.21
