# Supplementary material for: SAMHD1 is a key regulator of the lineage-specific response of acute lymphoblastic leukaemias to nelarabine
Source: Commun Biol. 2020 Jun 24;3:324. doi: 10.1038/s42003-020-1052-8 (PMC7314829; doi:10.1038/s42003-020-1052-8)
Supplement: Supplementary file 7 — Description of Additional Supplementary Files [file 42003_2020_1052_MOESM7_ESM.pdf]

Descriptions of Additional Supplementary Files

Supplementary Data 1. Gene transcripts differentially regulated (mRNA abundance) between T-ALL and B-ALL cell lines based on data derived from GDSC, CCLE, or CTRP.

Supplementary Data 2. Pathway analysis using the PANTHER database to identify differentially regulated processes based on genes differentially regulated between B-ALL and T-ALL cell lines in the CTRP, CCLE, and GDSC.

Supplementary Data 3. Genes whose expression is directly or inversely correlated with the nelarabine AUC based on CTRP data.

Supplementary Data 4. Genes whose expression is directly or inversely correlated with the nelarabine AUC based on CTRP data.

Supplementary Data 5. Data underlying graphs.
