# Supplementary material for: SAMHD1 is a key regulator of the lineage-specific response of acute lymphoblastic leukaemias to nelarabine
Source: Commun Biol. 2020 Jun 24;3:324. doi: 10.1038/s42003-020-1052-8 (PMC7314829; doi:10.1038/s42003-020-1052-8)
Supplement: Supplementary file 9 — Reporting Summary [file 42003_2020_1052_MOESM9_ESM.pdf]

nature research

Corresponding author(s): Martin Michaelis and Jindrich Cinatl

Last updated by author(s): Jun 2, 2020

Reporting Summary

Nature Research wishes to improve the reproducibility of the work that we publish. This form provides structure for consistency and transparency in reporting. For further information on Nature Research policies, see Authors & Referees and the Editorial Policy Checklist.

Statistics

For all statistical analyses, confirm that the following items are present in the figure legend, table legend, main text, or Methods section.

n/a	Confirmed	
☐	☒	The exact sample size (n) for each experimental group/condition, given as a discrete number and unit of measurement
☒	☐	A statement on whether measurements were taken from distinct samples or whether the same sample was measured repeatedly
☐	☒	The statistical test(s) used AND whether they are one- or two-sided<br>*Only common tests should be described solely by name; describe more complex techniques in the Methods section.*
☒	☐	A description of all covariates tested
☐	☒	A description of any assumptions or corrections, such as tests of normality and adjustment for multiple comparisons
☐	☒	A full description of the statistical parameters including central tendency (e.g. means) or other basic estimates (e.g. regression coefficient) AND variation (e.g. standard deviation) or associated estimates of uncertainty (e.g. confidence intervals)
☐	☒	For null hypothesis testing, the test statistic (e.g. F, t, r) with confidence intervals, effect sizes, degrees of freedom and P value noted<br>*Give P values as exact values whenever suitable.*
☒	☐	For Bayesian analysis, information on the choice of priors and Markov chain Monte Carlo settings
☒	☐	For hierarchical and complex designs, identification of the appropriate level for tests and full reporting of outcomes
☒	☐	Estimates of effect sizes (e.g. Cohen's d, Pearson's r), indicating how they were calculated

Our web collection on statistics for biologists contains articles on many of the points above.

Software and code

Policy information about availability of computer code

Data collection	GDSC methylation data - data for cell line methylation associated with the GDSC database (Iorio 2016) were obtained from the GEO website (series GSE68379 - https://www.ncbi.nlm.nih.gov/geo/query/acc.cgi?acc=GSE68379)
Data analysis	Illumina HumanMethylation450 BeadChip array data were preprocessed (Noob normalised) using R (version 3.6.1) Bioconductor (version 3.10) package Minfi (version 1.32.0) (Aryee 2014).

For manuscripts utilizing custom algorithms or software that are central to the research but not yet described in published literature, software must be made available to editors/reviewers. We strongly encourage code deposition in a community repository (e.g. GitHub). See the Nature Research guidelines for submitting code & software for further information.

Data

Policy information about availability of data

All manuscripts must include a data availability statement. This statement should provide the following information, where applicable:

- Accession codes, unique identifiers, or web links for publicly available datasets
- A list of figures that have associated raw data
- A description of any restrictions on data availability

All source data underlying the graphs, which are not based on publicly available data from CCLE, CTRP, GDSC, and patient gene expression data (Gene Expression Omnibus ID GSE66006), are presented in Supplementary Data 5. All other data are present in the manuscript or available from the corresponding authors upon reasonable request.

October 2018

Field-specific reporting

Please select the one below that is the best fit for your research. If you are not sure, read the appropriate sections before making your selection.

☒ Life sciences ☐ Behavioural & social sciences ☐ Ecological, evolutionary & environmental sciences

For a reference copy of the document with all sections, see nature.com/documents/nr-reporting-summary-flat.pdf

Life sciences study design

All studies must disclose on these points even when the disclosure is negative.

Sample size	We did not perform a sample size calculation. We analyzed data derived from databases, which was determined by data availability. Moreover, we used a substantial panel of 26 cell lines
Data exclusions	We have not excluded data.
Replication	All data represent at least three independent experiments.
Randomization	We analyzed data from databases and performed cell culture experiments. Hence, randomization was not necessary.
Blinding	We analyzed data derived from databases and performed cell culture experiments, which produced numerical read-outs. Hence, blinding was not necessary.

Reporting for specific materials, systems and methods

We require information from authors about some types of materials, experimental systems and methods used in many studies. Here, indicate whether each material, system or method listed is relevant to your study. If you are not sure if a list item applies to your research, read the appropriate section before selecting a response.

Materials & experimental systems

n/a	Involved in the study	
☐	☒	Antibodies
☐	☒	Eukaryotic cell lines
☒	☐	Palaeontology
☒	☐	Animals and other organisms
☒	☐	Human research participants
☒	☐	Clinical data

Methods

n/a	Involved in the study	
☒	☐	ChIP-seq
☒	☐	Flow cytometry
☒	☐	MRI-based neuroimaging

Antibodies

Antibodies used	SAMHD1 (Proteintech, 12586-1-AP, 1:1000), β-actin (BioVision, 3598R-100, 1:5000), pSAMHD1 (Cell Signaling, 89930S, 1:1000), GAPDH (Trevigen, 2275-PC-10C, 1:5000), IRDye®800CW Goat anti-Rabbit (LI-COR Biotechnology, 926-32211)
Validation	SAMHD1 (Proteintech, 12586-1-AP): by manufacturer, positive WB detected in HepG2 cells, human brain tissue, human heart tissue, K-562 cells, L02 cells, mouse heart tissue, KD/KO validated in several publications (https://www.ptglab.com/Products/SAMHD1-Antibody-12586-1-AP.htm#publications); β-actin (BioVision, 3598R-100,): by manufacturer, positive WB detected in human tissue lysate; pSAMHD1 (Cell Signaling, 89930S), by manufacturer, 1) Western blot analysis of extracts from THP-1 cells, untreated (-) or treated with calf intestinal phosphatase (CIP) plus λ phosphatase (+), 2) Western blot analysis of extracts from Jurkat and THP-1 cells, untreated (-) or treated with TPA (12-O-Tetradecanoylphorbol-13-Acetate) (80 nM, 16 hr; +), validated in 2 publications, 1) Shi, B., Sharifi, H. J., et al. (2018) 'Inhibition of HIV early replication by the p53 and ist downstream gene p21' Virology Journal 15(1):53, 2) Kinnetz, M., Alghamdi, F., et al. (2017) 'The impact of p53 on the early stage replication of retrovirus.' Virology Journal 14(1):151; GAPDH (Trevigen, 2275-PC-10C, 1:5000): validated in several publications, https://trevigen.com/products-services/cell-stress-and-dna-damage/apoptosis/cell-stress-and-dna-damage-apoptosis-antibodies/cell-stress-and-dna-damage-apoptosis-antibodies-anti-g3pdhgapdh/anti-g3pdh-human-polyclonal-antibody/

Eukaryotic cell lines

Policy information about cell lines

Cell line source(s)	DSMZ (Deutsche Sammlung von Mikroorganismen und Zellkulturen GmbH, Braunschweig, Germany), ATCC (Manassas, VA, US), Cambridge Enterprise Ltd. (Cambridge, UK)

Authentication	STR
Mycoplasma contamination	All cell lines tested negative for mycoplasma contamination
Commonly misidentified lines<br>(See ICLAC register)	We did not use cell lines known to be commonly misidentified.
